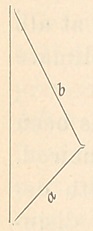# American Dental Society of Europe

**Published:** 1885-11

**Authors:** 


### AMERICAN DENTAL SOCIETY OF EUROPE.

THIRTEENTH ANNUAL MEETING, HELD IN BERLIN, AUGUST, 1885.

REPORTED EXPRESSLY FOR THE INDEPENDENT PRACTITIONER.

The meeting was opened by the reading of a paper from Dr. Du Bouchet, of Paris, on "The Treatment of Pulp Canals," of which the following is a summary :

The subject of pulp canals, their treatment and filling, occupies a large place in the discussions of our Dental Societies at home. The results obtained, good or bad, are promptly appreciated by the patient. It is interesting to note the various methods of treatment, displaying experience and considerable manipulative skill, in the attainment of the same end—immunity from pain, and durability. I will give as briefly as possible my several ways of proceeding, from the moment when we have obtained the partial devitalization of the pulp in order to prevent useless pain to the patient in its removal. Let us, for convenience, suppose an inferior cavity in the crown of a molar, from which we are about to extract the pulp. I would here say that I am a great believer in the exclusion of air and moisture as a necessary measure to success. Before my class at the *Institut Odontotechnique* of France, I have particularly insisted upon this condition, which to many seems to be one of the most difficult things to realize. The rubber dam being applied, we remove the pulp as thoroughly as possible by means of one of the various extractors, and dry the canals thoroughly by means of bibulous paper points. We next fill the canals loosely with these paper darts, the points of which are slightly moistened in old-fashioned wood creosote. The operation is completed by filling the pulp chamber with soft gutta-percha, and the rest of the cavity with a harder grade of the same article. If we find the paper points, after forty-eight hours, presenting no considerable degree of dampness and no other odor than that of creosote, we may replace them by fresh ones absolutely dry, and seal up the cavity. On the other hand, should we find upon the removal of our first filling that the paper points were considerably moistened or discolored, the canals should be dried with absolute alcohol, and allowed to remain several days filled with the paper twists. Should the periodontitis persist, I apply my aspirator, or artificial leech, preferring a slight derivation morning and evening to drawing a quantity of blood from the gum at a single sitting. I have found the instrument just mentioned of most efficient application in numerous instances. It is known to some as a modification of Dr. Wingate's Depurator. For a permanent root-filling, I insert as deeply as possible an adjusted splinter or slip of rattan wood, the extreme end of which has been dipped into wood creosote. By the side of this I introduce a fine thread of very soft gutta-percha, until I have filled the canals and half the pulp chamber, which latter I complete with oxy-phosphate cement. When the pulp canal is filled with putrescent matter I do not attempt to cleanse it thoroughly at the first sitting, but remove all debris in the body and near the apex of the canal, and thoroughly syringe out the cavity and canal with fresh oxygenated water, closing the canal at its entrance with only a pellet of cotton, rather loosely inserted. The directions given to the patient are the following : Every evening, before retiring and after having well brushed the teeth, syringe well the cavity of the tooth and root with oxygenated water, and close promptly with a cotton pellet. At the second sitting, which is generally upon the second or third day, I endeavor to clean the canal more thoroughly, and then I repeat the syringing, and closely stop the canal with cotton. This is repeated for several days, according to the nature of the case, after which the canal is treated as described above, when, if all goes smoothly the canals may be filled in a permanent way. In my hands this treatment has generally proved successful, although I am aware it is not quite the same as that followed by others of the profession.

DISCUSSION.

*Dr. Sachs*—In cases where I have destroyed and extirpated the pulp, I dry the canal with chloroform, alcohol, and hot air, and fill at once with gutta-percha threads. They easily go up to the apex of the root. I never use gutta-percha dissolved in chloroform, as I believe the latter, retained by the gutta-percha near the apex, will not evaporate, and the gutta-percha remains as a soft mass, which may cause periosteal trouble.

When a fistulous opening on the gum is present, I clean the pulp chamber and canal up to the apex, wash out with alcohol and chloroform, dry with hot air, and pump to the apex a little iodoform solution (iodoform 1, ether 10,) with a nerve-broach covered thinly with cotton, and fill the canal at once with gutta-percha, and the crown, if desirable, permanently. Such teeth, treated as described, very rarely give any trouble afterwards. In case the canals are filled with putrid matter, I clean the pulp-chamber and about one-half of the root canal at the first sitting, carefully avoiding any pressure which could drive putrid matter through the foramen. After syringing out with warm water, I use the hot air syringe freely, then very loosely put into the pulp-chamber and the clean part of the canal a piece of cotton dipped in the iodoform solution, and close the tooth for three or four days with a temporary stopping. At the second sitting I remove the filling and cotton, and try to clean the canal up as far as possible, but very gently, to prevent pressure, as before remarked, and wash out and treat as before. At the third sitting, if there is no odor or moisture in the canal, I fill with gutta-percha permanently, and finish.

*Dr. George*—Suggested that the readiness with which wood absorbs moisture might be an objection to the method detailed in the paper. He uses for difficult cases thin gold wire dipped in a solution of gutta-percha, but would prefer wood if it could be shown to be equally good.

*Dr. Patton*—My system for filling *such canals as can be filled*, is the old style of cotton wool and oxy-chloride of zinc, in the mixing of which I combine some of the iodoform crystals, incorporating the wool with the rather thin mixture and working it gradually up the canal into place. A pressure exerted from the body of the pulp-chamber causes the cement in this state to fill up any interstices not thoroughly occupied by the wool, and I am of the opinion, though I may be alone in it, that by this means the canals are filled. I have had very few failures, therefore I still adhere to it, and I have still to hear of the system which has *no* failures to record.

When the canals are so minute that it is impossible to fill them thoroughly, after cleaning out, as far as my manipulative capacity allows, I introduce the mixture of oxy-chloride of zinc and iodoform combined, somewhat thinner, and force it by pressure of a pellet of cotton until it will fill up what it can, trusting the rest to luck—the boon companion of dental medicaments in more treatments than this.

*Prof. Hollander*—It seems to me that the gentlemen have been mixing many things together. The question is not how shall I fill, but how shall I disinfect, root canals, and the method of proceeding must depend largely upon the conditions present, not in the canal alone, but around the root. There are, furthermore, as stated by Dr. Richter, many roots which it is impossible to treat at all. As for disinfectants, many are in use, and mercurial sublimate ranks very high among them.

I would not recommend iodoform in the manner which has been presented, nor should applications of it be frequently renewed. The mode of action of iodoform is peculiar. By contact with decomposing matter iodine is set free, which accomplishes the disinfection. This takes place very slowly, so that we do not get the full effect till many hours after application is made. When iodoform is employed for disinfecting a pulp-canal, it is not necessary that it should be brought quite to the apex. The free iodine will work its own way along the canal and effect a complete disinfection.

*Dr. Patton*—"There is nothing that succeeds like success." What we need is results, and I have had successful, satisfactory results from my practice. This is enough for me.

It is impossible to lay down fixed rules. The temperament and nervous organization of both patient and operator play an important role in practice. What is good for one is not good for another, even if the cases are quite similar. What one man can do under peculiar circumstances, another will fail to do in that particular manner, but each succeeds best in his own way; "experientia docet," or, as the Irishman said, "experience does it," and only time will teach a practitioner, after having studied his patients' mouths for some years, what is really the best to be done in each individual case. Every year proves to me more and more that an eclectic use of material is necessary, and the best but hardest things to find in the market are the substantial wares, judgment and common sense.

*Dr. Miller*—I would like to call the attention of the gentlemen to the paper points referred to by Dr. DuBouchet. Dr. Abbot taught me the use of these when I first began the practice of dentistry, and I have been using them ever since. I find them invaluable, and think I would nearly as soon be without nerve broaches as without these paper points. They are excellent for drying out root canals, and for carrying antiseptics to the apex of the root.

They must be made from fine, tough silk paper, and with a little practice in turning them they can be made so stiff as to pass easily to the apex of even crooked roots. They are also valuable in determining the course of fistulous tracks. The paper is cut for rolling in the form of the figure, held at the side *a*, between the thumb and index finger of the left hand, and rolled between the thumb and middle finger of the right hand, beginning at about the point *b*, and twisting towards the apex. The first attempt to make them will fail. Those who wish to make good paper points must try again. I am very much indebted to Dr. Galbreath, of Hanover, for showing me a very valuable use for a very simple and cheap instrument—the common Swiss watch broach. These broaches are very fine, three or four-sided, and slightly tapering to the point; they should be so soft as to easily bend without breaking. They are much finer than any broaches I ever saw in the dental depots.

For the finest canals I wind on one of these broaches just enough threads of cotton to carry the antiseptic, and it is very seldom indeed that one cannot reach the apex. If desired, by slightly loosening the cotton before introducing it, we may withdraw the broach, leaving the cotton in the canal. One word regarding the question of antiseptics. It appears that Dr. Hollander has, no doubt unintentionally, given the impression that iodoform acts only by setting iodine free, and that we cannot expect it to accomplish anything before twenty-four hours after application. This is, of course, a wrong impression. Iodoform has, independent of the presence or absence of putrifying matter, an immediate powerful antiseptic action, and it is this property which we make most use of in dental practice.

*Prof. Hollander*—I am obliged for the correction. I fear I did not express myself clearly on that point.

*Dr. Van Marter*—I use the broaches referred to by Prof. Miller, and find them very valuable.

### ON THE ARTIFICIAL PRODUCTION OF DENTAL CARIES.—BY DR. F. FOERSTER.

Many of the members of the American Dental Society of Europe here present will remember that at the meeting of this

Society in Cologne, August, 1882, Prof. Miller presented a paper on the agency of micro-organisms in the production of dental caries. In that paper the view was presented that while the phenomena connected with dental caries are often very puzzling, and do not as yet admit of a satisfactory explanation, yet, in the majority of cases, these phenomena must be attributed to the action of external agents, among which micro-organisms occupy the most important place. This view was supported by a demonstration of microscopic preparations of carious dentine, produced outside of the mouth.

Since that time Prof. Miller has continued his experiments, and has succeeded in producing artificial caries which is not to be distinguished from natural. At the same time, Milles and Underwood, in a lecture before the Odontological Society of Great Britain, stated that they repeated these experiments and had arrived at very different results, but as has been already pointed out, their experiment was made under such different and such abnormal conditions that negative results might have been predicted from the beginning.

There is no subject of more importance to practitioners of dentistry than the etiology of dental caries, and this fact alone fully justifies my presenting to you the results of my experiments on the production of caries outside of the human mouth. In these experiments I have endeavored to follow the rules at different times laid down in the INDEPENDENT PRACTITIONER.

A number of sound teeth, or teeth from which the carious portions had been thoroughly removed, were broken into pieces of various sizes, or sawed into sections about one mm. thick. These were placed in a mixture of bread and saliva of about the consistency of thick cream, then set in an incubator and kept at the temperature of the human body. In a few hours the mixture showed a very strong acid reaction, due, according to Miller, to the production of lactic acid. At the end of each week the broken teeth and sections were taken out of the mixture, washed in cold water, put into a fresh dish of saliva and bread, and replaced in the incubator. After the seventh week the dentine was so far softened that sections could be easily cut on the freezing microtome. These were stained in fuchsine, washed in absolute alcohol, cleared with oil of cloves, and mounted in Canada balsam. A microscopic examination showed that organisms had penetrated many of the tubules to a considerable depth. With each week the softening of the dentine became more extensive, and microscopic preparations, made from time to time, showed that the micro-organisms were penetrating deeper and deeper into the tissue of the tooth, until at the end of the fifth month the tubules were found to be much dilated and the walls broken down, in some places forming oval spaces or caverns in the dentine; in other words, showing all the characteristics of natural caries, and not to be distinguished from such. At the end of six months, when I ceased experimenting, I found the broken teeth and sections so soft that I could easily penetrate them with a broken excavator. Even the half molars could be pierced like cheese.

The enamel, which was entirely disintegrated, showed the soft, chalky, white color which we so often meet with on the buccal surfaces of molars near the gum, and could easily be picked from the dentine. At the same time a number of pieces of dentine from the same teeth were subjected to the action of fungi from the human mouth, but under such conditions that no acid could be found. These pieces remain unchanged to this day. The results of my experiments show that the first stage of dental caries is the decalcification of tooth-substance by acids, the greater quantity of which is, according to Miller, formed in the mouth by fermentation, and that it is highly improbable that the fungi of the human mouth can have any effect whatever upon teeth which have not to some degree been previously softened by the action of acids, and that the second stage is the simple destruction of the decalcified tooth-substance by micro-organisms.

Dr. Sachs read a paper on

THE DETERMINATION OF MATERIALS FOR FILLING TEETH.

of which the following is a summary:

Nothing in the practice of dentistry depends so much upon the taste, ability, prejudice and caprice of the operator, as the choice of material for filling a decayed tooth. In all the different handbooks we find no strict law when and in what places we should use the different materials employed for the purpose. The opinions of the most skillful and experienced dentists differ so widely, that the young man beginning to practice his profession finds himself in a difficult situation in regard to this point.

There is no material used for filling teeth which does not in certain cases promise the best results, while in others it would be worse than useless. Of the properties desirable in a filling material, gold possesses more than any other, and should always be employed when the structure is dense and hard, except in those instances in which, for æsthetic reasons, its use is contraindicated or objected to by the patient, or where the cavity is very difficult of access. It is an excellent material for filling approximal and lingual cavities in front teeth, or masticating buccal and approximal surfaces of molars. But it should never be used for filling soft, chalky teeth of young persons, as the work will soon fail, and secondary decay often be found in less than one year. Nor should it be used for filling teeth with very frail or thin walls, or for deciduous teeth.

Gold may be employed in many cases in connection with tin, as first recommended by Dr. Abbot, of Berlin. This combination has in certain cases a great advantage over gold or tin alone. It can be worked with great ease and rapidity, it is easier adapted to the walls of the cavity, it is not injured by moisture, and it expands slightly after insertion. These properties make it highly useful for filling soft, chalky teeth, or for the cervical walls of approximal cavities in molars and bicuspids. It may be used with success for crown and fissure fillings for children from six to fifteen years of age, for all approximal cavities excepting the six anterior teeth, for temporary and half erupted molars, in which we often find large fissures on the grinding surface.

One sheet of No. four to six non-cohesive gold is laid upon a sheet of tin-foil of about the same thickness, and cut in three strips, which must be twisted between the fingers into a rope. I prefer to have the tin-foil on the outside, having found it easier to manipulate in this form than when the gold is on the outside. The oxidation of the tin, which will take place after a short time, causes a very close union of the two metals. The filling then assumes a dark or gray color like amalgam, but it does not stain the tooth.

Tin alone forms a valuable material for filling teeth, but ever since its combination with gold has had such success, I should recommend it to students only for the sake of practice.

Amalgam has its advocates and opponents. The latter say that the use of amalgam has discredited dentistry, that the ease with which it can be worked has caused many dentists to do poorer work than they would otherwise have done. This may be true in many cases, but no one can doubt that thousands of teeth have been saved by the use of this material, even in the hands of quacks, who are not competent to make even a poor gold filling.

Amalgam has also proved very valuable for filling third, and distal surfaces of second molars. In cases where decay has gone very far below the margin of the gum in approximal cavities of molars and bicuspids, it is a good method to cover the cervical wall with amalgam and finish with gold. Of all the different amalgams in use, I prefer for filling that third of the distal approximal cavities in molars that lies near or below the gum, the common copper amalgam, as this has, theoretically and practically, been proved to prevent secondary decay better than the finest gold amalgam. The remaining part of the cavity must be filled with an amalgam that will oxidize as little as possible. If copper amalgam is inserted entirely free from moisture, it will not stain the tooth at all, but the metal itself will become very dark in color.

Gutta-percha and its different preparations may be used as a temporary filling to the greatest advantage.

Cement fillings may be used for cavities with exceedingly fragile, high walls, where it is not advisable to use amalgam, and where you do not wish to cut the walls down ; also for cavities where only one high wall remains, the others having broken away. An example of this we frequently see in bicuspids. It may also be used to fill tender and sensitive milk teeth, when you have no reason to suppose that the pulp is seriously affected. It may, furthermore, be used as a temporary filling for sensitive and soft upper front teeth, where the patient objects to the use of gold. It may also be used to fill the lower portion of very deep cavities; in these cases time is saved and the pulp is protected. But the most important instances in which cements may be used are for capping exposed or very thinly covered pulps, for filling the pulp cavity and canals of dead teeth, and for setting pivot teeth. The oxy-chlorides and oxy-phosphates should, however, never be used in proximal cavities, unless the neck of the tooth is protected by a layer of gutta-percha.

DISCUSSION.

*Prof. Miller*—Since Dr. Sachs has called attention to tin and gold fillings, I cannot let the opportunity go by without saying a word in their favor. The combination is now extensively used by Americans in Europe, and I have thus far met with no one who had given it a trial who does not speak in the highest terms of it.

The statement of Dr. Sachs, that it requires the same care to make a filling of tin and gold that it does of gold alone, is misleading. Of course, we should use equal care in the insertion of every filling, of whatever material, for the lack of care is liable to be followed by slovenly work, but a tin and gold filling will succeed with an amount of care small enough to wreck a gold filling. To illustrate: A molar of a child three to four years old is decayed on the grinding surface, and the child very restless. We dry the cavity, remove the debris and superficial caries only, saturate with carbolic acid and fill with tin and gold, without drying, in from two to four minutes, without pain, and the child wants us to fill another tooth. This filling will last longer than a dozen gold fillings, made with the same care. It is the ease of insertion, freedom from injury by moisture, slight expansion after filling, thus hermetically sealing the cavity, which render this combination so valuable. I find that I am going over the old story which I have told so often, and if you do not know it already you never will.

*Dr. Van Marter*—I was taught the use of tin and gold many years ago, by Dr. Abbot; I have used it very extensively, and like it very much.

(TO BE CONTINUED.)

## Figures and Tables

**Figure f1:**